# The Role of Preventive Pediatric Dentistry in Oral Health: An Evidence-Based Narrative Review: Short title: Preventive Pediatric Dentistry and Oral Health

**DOI:** 10.31661/gmj.v15i.4198

**Published:** 2026-08-03

**Authors:** Fouad Hassan Althobaiti, Maha Alsane, Myle Akshay Kiran, Tasneem Ambon, Latifah Fahad Almashabi, Rawan Ali Alqahtani, Noura Mohammed, Aljadaan, Atheer Haif Alotaibi, Sultan Abdullah Alotaibi, Haya Bin Osayl, Jawaher Mohammed Alsubaie, Roaa Saleh Aloraini, Abdulaziz Alribi, Nawaf Alyousef, Bader Ibrahim Alshehri

**Affiliations:** ^1^ Pediatric Dentistry Department, Ministry of Health, Taif, Saudi Arabia; ^2^ Collage of Dentistry, King Saud University, Riyadh, Saudi Arabia; ^3^ Department of Scientific Clinical Research and Pharmacology, Acharya Nagarjuna University, Guntur, India; ^4^ Medsravts in Education and Clinical research, Guntur, India

**Keywords:** Preventive Pediatric Dentistry, Early Childhood Caries, Fluoride, Dental Sealants, Silver Diamine Fluoride, Oral Health Promotion, Minimally Invasive Dentistry, Caries-Risk Assessment

## Abstract

Early childhood caries and other preventable oral diseases remain among the most common chronic conditions affecting children worldwide. Their burden is disproportionately concentrated among socially disadvantaged, rural, and medically vulnerable populations, where delayed dental attendance and limited access to preventive care contribute to disease progression, pain, infection, impaired nutrition, school absenteeism, and reduced quality of life. This narrative review summarizes contemporary evidence on preventive pediatric dentistry, with emphasis on caries-risk assessment, fluoride therapy, pit-and-fissure sealants, oral hygiene instruction, behavior modification, dietary counseling, anticipatory guidance, early dental visits, silver diamine fluoride, minimally invasive care, school-based programs, and emerging preventive technologies. Evidence consistently supports prevention-oriented, risk-based care models that combine professional interventions with caregiver education, dietary change, and community-level strategies. Fluoride exposure, sealant placement, motivational interviewing, and integrated medical-dental prevention are particularly important for high-risk children. Silver diamine fluoride and atraumatic or interim restorative approaches may reduce disease progression and improve access to care when conventional restorative treatment is difficult or unavailable. However, implementation remains limited by caregiver knowledge, socioeconomic inequality, workforce constraints, reimbursement barriers, and inconsistent integration of oral health into primary care systems. Preventive pediatric dentistry should therefore move beyond isolated clinical procedures toward coordinated, culturally responsive, evidence-based care that begins early in life and continues across childhood and adolescence.

## Introduction

Oral health is an integral component of general health, particularly during childhood, when dietary practices, hygiene behavior, microbial exposure, and family routines shape long-term disease risk. Dental caries remains one of the most prevalent noncommunicable diseases globally and is highly preventable when effective individual, family, clinical, and public-health interventions are implemented early [1, 2]. In children, untreated caries can cause pain, infection, sleep disturbance, impaired eating, poor growth, speech difficulties, reduced school attendance, and psychosocial distress. The consequences extend beyond the mouth and may impose substantial financial and caregiving burdens on families and health systems [2, 3].

The burden of pediatric oral disease is not distributed equally. Children from low-income households, rural communities, ethnic minority groups, and families with limited health literacy are more likely to experience early childhood caries, delayed dental attendance, and unmet treatment needs [1, 4]. These inequities reflect a combination of biological, behavioral, social, commercial, and health-system determinants, including frequent exposure to free sugars, limited access to fluoride, inadequate preventive services, and lower availability of child-centered dental care [1, 5]. As a result, effective prevention requires more than chairside instruction; it requires coordinated strategies that address family behavior, community environments, service delivery, and policy.

Preventive pediatric dentistry has therefore shifted from a restorative, disease-response model toward early, risk-based, and minimally invasive care. Modern prevention integrates caries-risk assessment, fluoride exposure, sealants, dietary counseling, caregiver engagement, anticipatory guidance, behavior-change techniques, school-based programs, and minimally invasive interventions such as silver diamine fluoride and atraumatic restorative treatment [3, 6, 13, 15]. This review aims to synthesize evidence-based strategies in preventive pediatric dentistry and to discuss implementation barriers, clinical implications, and future directions for improving oral health outcomes in children.

## Materials and Methods

### Study Design

This manuscript was structured as an evidence-based narrative review. A narrative review design was considered appropriate because the objective was to provide a clinically integrated overview of preventive pediatric dentistry rather than to estimate pooled intervention effects. The review synthesizes evidence from clinical guidelines, systematic reviews, randomized and non-randomized clinical studies, public-health reports, and relevant narrative reviews.

### Literature Search Strategy

A structured literature search was conducted in PubMed/MEDLINE, Scopus, and Google Scholar for peer-reviewed publications and authoritative guidance documents published primarily between January 2015 and April 2025. Additional seminal references published before 2015 were considered when they provided foundational evidence or were directly relevant to current preventive practice. Search terms included combinations of: pediatric dentistry, preventive dentistry, early childhood caries, dental caries prevention, fluoride varnish, fluoride toothpaste, community water fluoridation, dental sealants, silver diamine fluoride, atraumatic restorative treatment, motivational interviewing, dietary counseling, anticipatory guidance, caries-risk assessment, school dental programs, teledentistry, and oral health promotion.

### Eligibility Criteria

Eligible sources included English-language publications addressing preventive oral-health strategies for infants, children, or adolescents; studies evaluating clinical or public-health interventions for caries prevention or arrest; guidelines issued by recognized professional or public-health organizations; and systematic reviews or meta-analyses relevant to pediatric oral-health promotion. Publications were excluded if they focused exclusively on adult populations, were unrelated to prevention, lacked scientific relevance to pediatric dentistry, were unavailable in English, or provided insufficient methodological or clinical information.

### Data Extraction and Synthesis

Information was extracted on study type, population, preventive intervention, target age group, mechanism of action, clinical outcomes, implementation context, and reported limitations. Because of heterogeneity in study designs, populations, intervention protocols, and outcome measures, findings were synthesized narratively rather than statistically pooled. The synthesis prioritized higher-level evidence, including clinical practice guidelines, systematic reviews, randomized controlled trials, and major public-health reports. Where evidence was preliminary or inconsistent, this uncertainty was explicitly acknowledged.

## Etiology and Risk Factors of Pediatric Oral Diseases

Pediatric oral diseases, particularly dental caries, arise from complex interactions among biological, behavioral, environmental, and social determinants. Dental caries is a biofilm-mediated, sugar-driven, multifactorial disease characterized by repeated episodes of enamel and dentin demineralization. Frequent intake of fermentable carbohydrates promotes acid production by cariogenic biofilms, reducing plaque pH and shifting the oral environment toward mineral loss [3]. This process is intensified when salivary protection, fluoride exposure, plaque control, and dietary regulation are inadequate.

Biological risk factors include enamel defects, deep pits and fissures, reduced salivary flow, high levels of cariogenic microorganisms, and previous caries experience. Behavioral factors include infrequent toothbrushing, inadequate use of fluoridated toothpaste, limited parental supervision, frequent snacking, nocturnal bottle use, and high intake of sugar-sweetened beverages [3, 5]. Social determinants, including poverty, food insecurity, limited access to dental services, low caregiver health literacy, and cultural beliefs regarding primary teeth, further influence disease onset and progression [1, 4].

Risk-based pediatric dentistry therefore requires structured caries-risk assessment rather than uniform preventive advice for all children. Caries Management by Risk Assessment and other risk-based frameworks support identification of children requiring intensified fluoride exposure, dietary counseling, closer recall, sealants, silver diamine fluoride, or minimally invasive management [6]. Preventive care should be individualized according to risk level, age, developmental stage, family capacity, and access to dental services.

## Evidence-Based Preventive Strategies in Pediatric Dentistry

The main evidence-based preventive strategies are summarized in Table1.

**Table:** Table1. Evidence-Based Preventive Strategies in Pediatric Dentistry.

Preventive Strategy	Primary Mechanism	Target Population	Clinical Implication
Fluoride toothpaste and varnish	Enhances remineralization, reduces demineralization, and inhibits bacterial metabolism.	Infants, children, and adolescents; intensified use for moderate- and high-risk children.	Core preventive intervention; frequency and professional application should be guided by caries risk.
Dental sealants	Creates a physical barrier over pits and fissures, limiting plaque and carbohydrate retention.	Children and adolescents with erupting or fully erupted primary or permanent molars at risk.	Highly effective for occlusal caries prevention and arrest of selected non-cavitated lesions.
Dietary counseling	Reduces frequency of free-sugar exposure and acidogenic biofilm activity.	All children, especially those with frequent snacking, sugary drinks, or early childhood caries risk.	Should be family-centered, feasible, culturally sensitive, and reinforced over time.
Oral hygiene instruction and behavior modification	Improves plaque control and supports sustained fluoride exposure through supervised brushing.	Children and caregivers; tailored by developmental stage and health literacy.	Most effective when combined with motivational interviewing and caregiver engagement.
Anticipatory guidance and early dental visits	Identifies risk early and provides age-specific prevention before disease progression.	Infants, toddlers, and caregivers; ideally beginning within the first year of life.	Supports establishment of a dental home, early intervention, and caregiver education.
Silver diamine fluoride	Combines antimicrobial silver effects with fluoride-mediated remineralization.	High-risk children, young or anxious children, children with special healthcare needs, and underserved groups.	Useful for caries arrest; requires informed consent because of permanent black staining.
Minimally invasive care	Preserves tooth structure, stabilizes lesions, and reduces need for invasive treatment.	Children with active lesions, limited cooperation, or restricted access to conventional care.	Best used as part of comprehensive disease control, not as a stand-alone restorative substitute.
School- and community-based programs	Delivers prevention to populations with limited access to clinic-based dental care.	Children in underserved, rural, or high-risk communities.	Can reduce disparities when linked to referral pathways and sustained public-health support.

SDF: silver diamine fluoride.

### Fluoride Therapy

Fluoride remains the cornerstone of caries prevention because it enhances remineralization, reduces demineralization, and inhibits bacterial metabolism within dental biofilm [7]. Twice-daily use of age-appropriate fluoridated toothpaste is among the most practical and cost-effective preventive measures for children. Professional fluoride varnish provides additional benefit for children at moderate or high caries risk, particularly when applied at intervals determined by individualized risk assessment [7, 8]. Community water fluoridation continues to be an important population-level intervention where feasible and acceptable. Contemporary evidence indicates that fluoridation can reduce caries experience, although effect sizes may vary according to baseline fluoride exposure, socioeconomic context, availability of fluoridated toothpaste, and study period [9]. For children at high risk, prevention is strongest when fluoride toothpaste, professional fluoride varnish, dietary counseling, and regular recall are combined rather than delivered as isolated interventions.

### Dental Sealants

Dental sealants are evidence-based preventive interventions for occlusal surfaces of permanent molars and, where indicated, primary molars. They create a physical barrier that protects pits and fissures from plaque accumulation and fermentable carbohydrate exposure. Guidelines support sealant use for preventing and arresting non-cavitated pit-and-fissure carious lesions in children and adolescents [10]. Sealants are particularly relevant during the eruption of first and second permanent molars, when incomplete eruption, immature enamel, and limited brushing effectiveness increase caries susceptibility. Resin-based sealants generally demonstrate strong retention and caries-preventive effectiveness when placed under adequate moisture control. Glass-ionomer sealants may be useful where isolation is difficult or fluoride release is desirable, although retention profiles differ [10, 11]. School-based sealant programs are especially valuable for reducing disparities because they reach children who may not routinely access dental clinics [12].

### Oral Hygiene Instruction and Behavior Modification

Effective oral hygiene depends on both technical instruction and sustained behavior change. Children require developmentally appropriate toothbrushing guidance, use of fluoridated toothpaste in recommended amounts, and caregiver supervision until manual dexterity and self-regulation are sufficient. For younger children, caregiver brushing or assisted brushing is essential because independent brushing is often ineffective [6, 7]. Traditional didactic education alone is often insufficient to produce durable behavior change. Motivational interviewing, goal setting, tailored feedback, visual aids, and culturally responsive counseling may improve caregiver engagement and adherence to preventive practices [18]. Preventive messages should be reinforced during recall visits and adapted to the child’s developmental stage, family routines, language, health literacy, and socioeconomic context. Dietary Counseling Dietary behavior is central to caries prevention. The frequency, timing, and form of free-sugar exposure are particularly important because repeated sugar intake sustains acidogenic biofilm activity and prolongs periods of enamel demineralization [3, 5]. Early childhood caries is strongly associated with frequent snacking, sugar-sweetened beverages, sweetened liquids in bottles or sippy cups, and nighttime feeding practices that expose teeth to fermentable carbohydrates for prolonged periods [5]. International guidance recommends reducing free-sugar intake to less than 10% of total energy intake, with additional health benefits expected when intake is reduced below 5% [5]. In clinical practice, this recommendation should be translated into feasible family advice, such as limiting sugar-containing snacks between meals, avoiding sweetened beverages at bedtime, encouraging water as the main drink, and selecting healthier snacks. Dietary counseling should be supportive rather than blame-oriented, particularly for families facing food insecurity, cultural dietary norms, or limited access to healthy foods.

### Anticipatory Guidance and Early Dental Visits

Anticipatory guidance is a proactive, age-specific approach that prepares caregivers for predictable oral-health needs across developmental stages. It includes counseling on teething, oral hygiene, fluoride exposure, feeding practices, injury prevention, non-nutritive sucking habits, eruption patterns, and the importance of establishing a dental home [6]. Early dental attendance allows timely risk assessment, caregiver education, detection of early lesions, and initiation of minimally invasive preventive care. Integration of oral health into pediatric primary care is important because many infants and toddlers visit physicians before they establish regular dental attendance. Collaboration among pediatric dentists, general dentists, pediatricians, nurses, dietitians, schools, and community health workers can expand prevention, especially in underserved populations [1, 4].

## Special Preventive Interventions

### Silver Diamine Fluoride

Silver diamine fluoride (SDF) is a topical cariostatic agent with antimicrobial and remineralizing properties. Silver ions suppress cariogenic bacteria, while fluoride promotes remineralization of demineralized enamel and dentin [13]. SDF is particularly useful for young children, anxious patients, children with special healthcare needs, and populations with limited access to conventional restorative treatment. Clinical evidence supports the use of 38% SDF for arresting dentinal caries lesions in primary teeth, although outcomes vary by lesion activity, application frequency, oral hygiene, diet, and follow-up duration [13, 14]. The procedure is simple, inexpensive, noninvasive, and generally well tolerated. Its principal limitation is permanent black staining of arrested carious dentin, which may be unacceptable for some caregivers, particularly in anterior teeth. Informed consent is therefore essential and should include discussion of staining, expected benefits, need for follow-up, and possible subsequent restorative care [14].

### Minimally Invasive Restorative and Rehabilitative Approaches

Minimally invasive dentistry aims to preserve tooth structure, control disease activity, reduce treatment anxiety, and avoid unnecessary pulpal exposure. Atraumatic restorative treatment, interim therapeutic restorations, selective caries removal, and bioactive restorative materials can be valuable for children who cannot tolerate conventional restorative care or who are treated in community-based settings [15, 16]. Atraumatic restorative treatment using high-viscosity glass-ionomer cement can arrest caries and restore function while releasing fluoride and minimizing the need for rotary instrumentation [15]. Interim therapeutic restorations may stabilize lesions until definitive care is feasible. Selective caries removal should be guided by lesion depth, pulpal status, restorative objectives, and caries risk, with the goal of maintaining pulp vitality and minimizing overtreatment [16]. These interventions should not be viewed as substitutes for prevention; rather, they are best integrated into comprehensive caries-control plans. Emerging Preventive Technologies Emerging technologies in pediatric preventive dentistry include bioactive materials, resin infiltration, salivary diagnostics, microbiome-informed risk assessment, artificial intelligence-assisted screening, teledentistry, and laser-assisted approaches. Bioactive restorative materials may release calcium, phosphate, and fluoride ions and support remineralization in selected clinical situations [17]. Resin infiltration can be useful for non-cavitated proximal and smooth-surface enamel lesions by occluding lesion porosities and slowing progression without conventional cavity preparation [17]. Despite promising laboratory and early clinical findings, many emerging technologies require stronger long-term clinical evidence, standardized protocols, cost-effectiveness evaluation, and implementation research before routine adoption as standard preventive care [19]. Claims about these technologies should therefore remain cautious and should distinguish established clinical practice from investigational or adjunctive approaches.

### Challenges and Barriers in Implementing Preventive Pediatric Dentistry

Implementation of preventive pediatric dentistry is limited by patient-, caregiver-, provider-, system-, and policy-level barriers. Patient and caregiver barriers include dental anxiety, limited knowledge of oral-health risks, low perceived importance of primary teeth, competing family priorities, language barriers, and difficulty sustaining dietary and hygiene changes. Socioeconomic constraints may limit access to fluoride products, healthy foods, transportation, and dental appointments [1, 4]. Provider-level barriers include limited training in behavior change, motivational interviewing, pediatric risk assessment, and minimally invasive care. Time pressures, high patient volume, and reimbursement models that favor restorative treatment over prevention may further reduce preventive service delivery [20]. System-level barriers include shortages of pediatric dental providers, inadequate insurance coverage, weak integration of dental and medical care, limited school-based prevention, and unequal access in rural or underserved communities [1, 20]. Addressing these barriers requires multilevel strategies: caregiver-centered counseling, culturally appropriate education, risk-based recall systems, school-based fluoride and sealant programs, teledentistry, workforce training, medical-dental integration, and policy reforms that reimburse preventive services adequately. Sustainable improvements are most likely when clinical prevention is embedded within broader public-health and primary-care systems.

## Discussion

This review highlights that preventive pediatric dentistry is most effective when delivered as an integrated, risk-based model rather than as a collection of isolated procedures. Fluoride therapy, sealants, dietary counseling, oral hygiene support, and early dental visits are well-established components of caries prevention [6, 7, 8, 9, 10, 11, 12]. Their effectiveness is strengthened when preventive recommendations are individualized according to caries risk, caregiver capacity, developmental stage, and social context.

The mechanistic rationale for prevention is clear. Caries develops when the oral biofilm is repeatedly exposed to fermentable carbohydrates, producing acids that drive mineral loss. Fluoride shifts the balance toward remineralization; sealants protect anatomically susceptible surfaces; dietary counseling reduces acid challenges; and oral hygiene reduces biofilm accumulation while maintaining fluoride exposure [3, 7, 10]. These interventions therefore act on complementary stages of the disease pathway. For high-risk children, combining strategies is more biologically and clinically plausible than relying on any single preventive measure.

SDF and minimally invasive approaches have expanded the preventive and disease-control options available for young children and underserved populations. SDF is especially valuable where conventional restorative care is delayed, poorly tolerated, or inaccessible [13, 14]. Similarly, atraumatic restorative treatment and interim therapeutic restorations can stabilize disease while preserving tooth structure and reducing procedural burden [15, 16]. However, these approaches require appropriate case selection, caregiver communication, follow-up, and integration with long-term prevention.

The review also underscores the importance of social determinants and implementation science. Even highly effective interventions may fail when families cannot afford dental visits, obtain fluoride products, access healthy foods, or attend regular recall appointments. School-based sealant programs, fluoride varnish initiatives, medical-dental integration, and teledentistry can reduce access barriers, but they require sustainable financing, trained personnel, referral systems, and policy support [12, 20, 22, 24].

Emerging technologies may further improve prevention, but they should be interpreted cautiously. Bioactive materials, resin infiltration, laser-assisted protocols, digital risk prediction, and microbiome-based diagnostics are promising, yet evidence varies considerably in quality and maturity [17, 19, 23]. Broader public-health action is also required because oral diseases remain a major global health challenge across the life course [21, 25].

## Strengths and Limitations

A strength of this review is its broad synthesis of clinical, behavioral, and public-health strategies relevant to preventive pediatric dentistry. The manuscript integrates established interventions with newer minimally invasive and digital approaches while emphasizing equity, caregiver engagement, and risk-based care.

The main limitation is the narrative design. The review did not include PRISMA-based screening, duplicate study selection, formal risk-of-bias assessment, evidence grading, or meta-analysis. Therefore, it cannot provide pooled effect estimates or definitive comparative effectiveness conclusions. In addition, heterogeneity among study designs, populations, interventions, and outcomes limits direct comparison across preventive strategies. Future work should include systematic reviews and implementation studies evaluating long-term clinical outcomes, cost-effectiveness, acceptability, and scalability in diverse pediatric populations.

## Conclusion

Preventive pediatric dentistry is central to reducing the burden of childhood oral disease and promoting lifelong oral health. Evidence supports early, risk-based, family-centered care that combines fluoride exposure, sealants, dietary counseling, supervised oral hygiene, anticipatory guidance, and timely dental attendance. SDF and minimally invasive approaches provide additional options for caries control, particularly in young, anxious, medically vulnerable, or underserved children. Persistent barriers related to caregiver knowledge, socioeconomic inequality, workforce capacity, reimbursement, and medical-dental fragmentation must be addressed through integrated clinical, community, and policy strategies. Future progress will depend on culturally responsive prevention, stronger implementation systems, and careful evaluation of emerging technologies before broad clinical adoption.

## Conflict of Interest

The authors declare no conflict of interest.

## AI Disclosure Statement

During the preparation of this work, the author(s) used ChatGPT to assist with language editing and copyediting. After using this tool, the author(s) reviewed and edited the content as needed and take(s) full responsibility for the content of the publication. [CE3: Please confirm whether this AI disclosure is accurate according to GMJ policy.]
